# Chemotherapy and quality of life in advanced NSCLC – reply

**DOI:** 10.1054/bjoc.2001.1852

**Published:** 2001-07

**Authors:** Heather Anderson, Jim Carmichael, Marianne Nicolson, Penny Hopwood, Nick Thatcher

**Affiliations:** Chest Clinic, NW Lung Centre, Wythenshawe Hospital, Southmoor Road, Wythenshawe, Manchester M23 9LT


*British Journal of Cancer* (2001) **85**(1), 137–140
© 2001 Cancer Research Campaign
doi: 10.1054/ bjoc.2001.1851, available online at http://www.idealibrary.com on IDEAL®

### Chemotherapy and quality of life in advanced NSCLC

**Sir,**

The study reported by Anderson et al (2000) in the August issue of the *BJC* demonstrated a sustained improvement in symptom score with corresponding improvement in QOL, when gemcitabine (GC) was combined with best supportive care (BSC) vs BSC alone in advanced NSCLC. Although GC produced an objective response rate of 19%, with fewer patients requiring radiotherapy (RT); and the time to RT salvage was longer with GC, there was no difference in survival. While this study demonstrates once again that patients' symptoms and QOL do benefit from palliative chemotherapy in advanced NSCLC, it does not eliminate one important factor: the placebo effect associated with receiving intravenous chemotherapy.

It is common experience that patients' expectations from chemotherapy are greater than those of oncology professionals, and therefore patients are more willing to undergo chemotherapy even for small gain (Slevin et al, 1990). The very act of receiving anticancer therapy gives patients a sense of optimism associated with the perception that 'something active is being done' and that they are not just 'wasting away, waiting for the inevitable'. These factors can be very powerful psychological stimuli which lead patients to under-report symptoms either because they truly feel better (placebo effect) or because of underlying fear that reporting toxicity or symptomatic deterioration may lead to early cessation of the treatment.

Where the margin of benefit is so narrow, as in the case of this study of single agent GC in NSCLC, we feel that blinded placebo control trials are required to address the true benefit of cytotoxic chemotherapy in terms of symptomatic improvement and QOL.

De Deyn and D'Hooge (1996), in debating the ethical issues around placebo-controlled trials stated that for such studies to be considered ethical, it was important that no adequate therapy for the disease should exist and/or the presumed active treatment should have side effects. One realizes that the scenario reported by Anderson et al in their study fits De Deyn's criterion rather well.

While we may debate what constitutes an acceptable placebo in such a study, an appropriate 'placebo' may be the same agent given at a sub-therapeutic dose. The placebo arm would then require the same degree of monitoring as the treatment arm in order to confirm that no biological effect is observed on marrow, renal and hepatic function. Only then can we truly evaluate the effect of therapeutic doses of any chemotherapeutic agent on symptoms and QOL, free from observer and patient bias as long as the study remains blinded. And if such a study were to demonstrate equivalence in terms of symptom and QOL benefit, it would have a significant impact on our interpretation of similar studies where chemotherapy with BSC have been compared to BSC alone. Maybe we should use the window of opportunity provided by advanced NSCLC to set up such a study before the opportunity is lost for good.

**D Farrugia**
**Consultant Medical Oncologist,**
Cheltenham General Hospital,
Sandford Road,
Cheltenham, GL53 7AN, UK

**R Lewis**
**Consultant Respiratory Physician,**
Worcester Royal Infirmary,
Castle Street Branch,
Worcester, WR1 3AS

### REFERENCES

Anderson H (2000) Gemcitabine plus best supportive care (BSC) vs BSC in inoperable non-small cell lung cancer – a randomized trial with quality of life as the primary outcome. *Br J Cancer* **83**: 447–453, doi: 10.1054/bjoc.2000.1307

De Deyn P and D'Hooge R (1996) Placebos in clinical practice and research. *J Med Ethics* **22**: 140–146

Slevin M (1990) Attitudes to chemotherapy: comparing views of patients with cancer with those of doctors, nurses and general public. *Br Med J* **300**: 1458–1460

doi: 10.1054/ bjoc.2001.1852, available online at http://www.idealibrary.com on IDEAL®

### Chemotherapy and quality of life in advanced NSCLC – reply

**Sir,**

There are now 5 recent randomized trials assessing the value of chemotherapy versus best supportive care in chemotherapy naive patients with locally advanced or metastatic non-small cell lung cancer. The chemotherapy regimens were single agent gemcitabine, paclitaxel, vinorelbine in a selected elderly population, the combination mitomycin C, ifosfamide and cisplatin (MIC) and single agent docetaxel (Cullen et al, 1999; the Elderly Lung Cancer Vinorelbine Italian Study Group, 1999; Anderson et al, 2000; Ranson et al, 2000; Roszkowski et al, 2000). There was evidence of median or 1 year survival advantage in all studies except the gemcitabine study. However, we note that in the gemcitabine study more patients were WHO performance status 2 (72%) than in the other studies (18–32%), probably because asymptomatic patients were not eligible for entry into our study.

All of the studies have assessed quality of life – a difficult area of research, but all studies have shown some quality of life benefits with chemotherapy. In addition, our study was designed with quality of life as a primary outcome and criteria for sustained improvement in quality of life were established prior to data analysis, emphasizing the validity of our reported findings.

A recent report from the ECOG group (study E1594) showed that modern combination chemotherapies produced equivalent survival at one year (31–36%) for platinum combinations with taxol, gemcitabine, and docetaxel (Schiller et al, 2000). This one-year survival is better than the approximately 15% reported in the best supportive care groups in meta-analysis (Souquet et al, 1993; Marino et al, 1994; NSCLCCG, 1995). The ECOG group stopped treating WHO performance status 2 patients because of their poorer outcome and less than 7% patients in their study were in this category. This patient group needs to be the subject of further data analysis.

Perhaps we can now formulate treatment plans based on stage and performance status. Most oncologists treating patients with inoperable non-small cell lung cancer would accept a cisplatin-based regimen as standard therapy for metastatic disease especially for performance status 0 and 1 patients. For these small survival advantages quality of life improvements are important. Oncologists stop chemotherapy after 2 courses if the patient has not benefited.

We understand the authors may question the value of a new agent used alone in the symptom management of patients with non-small cell lung cancer. Patients entered into our trial should have received the best symptomatic therapy available. The authors question if a placebo effect with gemcitabine improved the patients' quality of life but provide no evidence to support this suggestion, nor do they support their contention that 'powerful psychological stimuli lead patients to under-report symptoms' whilst on chemotherapy. In both arms of our study improvement and deterioration in symptoms occurred but there was an overall advantage for patients treated with chemotherapy.

Placebo studies in cancer chemotherapy are hard to justify for the following reasons:

1. Asking a patient to attend for regular intravenous placebo injections is very problematic and considered unethical by some people.
2. The placebo cannot be blinded as the professional staff will know which patients are on the active drug because of side effects.
3. The authors' suggestion about using sub-therapeutic doses of active drugs as a placebo is unethical as toxicity would occur. Phase I trials are conducted to determine the recommended treatment dose for a given schedule dependent upon the maximum tolerated dose of drug allowing for toxicity. This dose is then used in phase II trials to confirm efficacy.
4. Professionals who regularly treat lung cancer would find it unethical to conduct a placebo-based study because of the published benefits with chemotherapy either in combination or as single agent therapy.
5. In trials patients who have unexpected side effects or progression stop therapy. In this trial the median number of cycles was 3 (i.e. 3 months). The benefits on gemcitabine vs BSC on quality of life were maintained at 4 months.
6. In this trial one may have expected a placebo response to include relief of dyspnoea but this symptom was better palliated by BSC at the 2 month assessment.
7. Randomization should reduce the tendency for a 'placebo response' with chemotherapy (if such a response exists), since patients with strong expectations or beliefs about the value of chemotherapy would have refused randomization.
8. A recent study assessing the influence of pre-treatment optimism on quality of life and progression-free survival after radical radiotherapy for lung cancer has shown no significant difference in 2 year survival (Ball et al, 2000). i.e. 'Patient optimism is not enough'.

**Heather Anderson, Jim Carmichael, Marianne Nicolson, Penny Hopwood, Nick Thatcher**

Chest Clinic, NW Lung Centre,
Wythenshawe Hospital, Southmoor Road,
Wythenshawe, Manchester M23 9LT

### REFERENCES

Anderson H, Hopwood P, Stephens RJ, Thatcher N, Cottier B, Nicolson M, Milroy R, Maughan TS, Falk S, Bond MG, Burt PA, Connolly CK, McIllmurray MB, Carmichael J on behalf of the UK NSCLC Gemcitabine Group (2000) Gemcitabine plus best supportive care (BSC) vs BSC in inoperable non-small cell lung cancer – a randomised trial with quality of life as the primary outcome. *Brit J Cancer* **83**(4): 447–453

Ball D, Borland R, Smith J, Bishop J, O'Brien P, Davis S, Olver I, Walker Q, Ryan G and Joseph D (2000) The influence of pre-treatment optimism on outcome in patients given radical radiotherapy for non-small cell lung cancer. *Lung Cancer* **29**(suppl 1): 173, abst 585

Cullen MH, Billingham LJ, Woodroffe CM, Chetiyawardana AD, Gower NH, Joshi R, Ferry DR, Rudd RM, Spiro SG, Cook JE, Trask C, Bessell E, Connolly CK, Tobias J and Souhami RL (1999) Mitomycin, ifosfamide and cisplatin in unresectable non small cell lung cancer: effects on survival and quality of life. *J Clin Oncol* **17**(10): 3188–3194

Marino P, Pampallona S, Preatoni A, Cantoni A and Invernizzi F (1994) Chemotherapy vs supportive care in advanced non small cell lung cancer. Results of a meta-analysis of the literature. *Chest* **106**: 861–865

Non Small Cell Lung Cancer Collaborative Group (1995) Chemotherapy in non small cell lung cancer: a meta-analysis using updated data on individual patients from 52 randomised clinical trails. *BMJ* **311**: 899–909

Ranson M, Davidson N, Nicolson M, Falk S, Carmichael J, Lopez P, Anderson H, Gustafson N, Jeynes A, Gallant G, Washington T and Thatcher N (2000) Randomised trial of paclitaxel plus best supportive care versus best supportive care for patients with advanced non small cell lung cancer. *J Natl Cancer Inst* **92**(13): 1074–1080

Roszkowski K, Pluzanska A, Krzakowski M, Smith AP, Saigi E, Aasebo U, Parisi A, Tran NP, Olivares R and Berille J (2000) A multicentre, randomised, phase III study of docetaxel plus best supportive care in chemotherapy naive patients with metastatic or nonresectable localised non small cell lung cancer. *Lung Cancer* **27**: 145–147

Schiller JH, Harrington D, Sandler A, Belani C, Langer C, Krook J and Johnson DH (2000) A randomised phase III trial of four chemotherapy regimens in advanced non-small cell lung cancer. *Proc Am Soc Clin Oncol* **19**: abst 2

Souquet PJ, Chauvin F, Boissel JP, Cellerino R, Cormier Y and Ganz PA (1993) Polychemotherapy in advanced non small cell lung cancer: a meta-analysis. *Lancet* **342**: 19–21

The elderly lung cancer vinorelbine Italian study group (1999) Effects of vinorelbine on quality of life and survival of elderly patients with advanced non-small cell lung cancer. *J Natl Cancer Inst* **91**(1): 66–72

*© 2001 Cancer Research Campaign*

